# Potential dynamic regional brain biomarkers for early discrimination of autism and language development delay in toddlers

**DOI:** 10.3389/fnins.2022.1097244

**Published:** 2023-01-09

**Authors:** Shengjuan Wu, Zhi Wen, Wenzhong Yang, Chengcheng Jiang, Yurong Zhou, Zhiwei Zhao, Aiqin Zhou, Xinglian Liu, Xiaoyan Wang, Yue Wang, Hong Wang, Fuchun Lin

**Affiliations:** ^1^Department of Child Health Care, Maternal and Child Health Hospital of Hubei Province, Tongji Medical College, Huazhong University of Science and Technology, Wuhan, China; ^2^Department of Radiology, Renmin Hospital of Wuhan University, Wuhan, China; ^3^Department of Radiology, Maternal and Child Health Hospital of Hubei Province, Tongji Medical College, Huazhong University of Science and Technology, Wuhan, China; ^4^Department of Radiology, Zhongnan Hospital of Wuhan University, Wuhan, China; ^5^State Key Laboratory of Magnetic Resonance and Atomic and Molecular Physics, National Center for Magnetic Resonance in Wuhan, Wuhan Institute of Physics and Mathematics, Innovation Academy for Precision Measurement Science and Technology, Chinese Academy of Sciences, Wuhan, China

**Keywords:** autism spectrum disorder, language development delay, dynamic regional homogeneity, dynamic amplitude of low-frequency fluctuation, early diagnosis

## Abstract

**Background:**

The early diagnosis of autism in children is particularly important. However, there is no obvious objective indices for the diagnosis of autism spectrum disorder (ASD), especially in toddlers aged 1–3 years with language development delay (LDD). The early differential diagnosis of ASD is challenging.

**Objective:**

To examine differences in the dynamic characteristics of regional neural activity in toddlers with ASD and LDD, and whether the differences can be used as an imaging biomarker for the early differential diagnosis of ASD and LDD.

**Methods:**

Dynamic regional homogeneity (dReHo) and dynamic amplitude of low-frequency fluctuations (dALFF) in 55 children with ASD and 31 with LDD, aged 1–3 years, were compared. The correlations between ASD symptoms and the values of dReHo/dALFF within regions showing significant between-group differences were analyzed in ASD group. We further assessed the accuracy of dynamic regional neural activity alterations to distinguish ASD from LDD using receiver operating characteristic (ROC) analysis.

**Results:**

Compared with the LDD group, the ASD group showed increased dReHo in the left cerebellum_8/Crust2 and right cerebellum_Crust2, and decreased dReHo in the right middle frontal gyrus (MFG) and post-central gyrus. Patients with ASD also exhibited decreased dALFF in the right middle temporal gyrus (MFG) and right precuneus. Moreover, the Childhood Autism Rating Scale score was negatively correlated with the dReHo of the left cerebellum_8/crust2 and right cerebellum_crust2. The dReHo value of the right MFG was negatively correlated with social self-help of the Autism Behavior Checklist score.

**Conclusion:**

The pattern of resting-state regional neural activity variability was different between toddlers with ASD and those with LDD. Dynamic regional indices might be novel neuroimaging biomarkers that allow differentiation of ASD from LDD in toddlers.

## 1. Introduction

Autism spectrum disorder (ASD) is a heterogeneous neurodevelopmental disorder, with varying clinical manifestations and disease severity. The prevalence is 1 in 44 in America,^1^ whereas approximately 1% in China (Sun et al., 2019). The core defects of ASD are impaired social functions, impaired language communication, rigid and repetitive behavior. Among them, language retardation may be an early concern for ASD children (Chung et al., 2011).

There are two difficulties in early diagnosis of ASD with language retardation. On the one hand, around 1–3 years old, the onset and severity of language retardation vary greatly in ASD, and there are usually no core symptoms. When language development is delayed, the score on Autism Diagnosis Scale will increase, which makes ASD and language development delay (LDD) easy to be confused. On the other hand, the prognosis of intervention varies greatly between ASD and LDD. For example, with appropriate intervention, LDD children who are diagnosed at the age of 2 years can reach typically developing peers at the age of 3–4 years (Schulte-Korne, 2014). In contrast, the prognosis of ASD is often worse than that of LDD, which involves life-long and continuous intervention and rehabilitation. Therefore, it is important to explore new indicators to distinguish ASD from LDD in the early stage.

Although lack of neurologic anatomical anomalies in conventional imaging, advanced imaging studies demonstrated that ASD is closely related to the structural and functional changes of the “social brain” (Amaral et al., 2008). The literature review about structural MRI studies reported the prefrontal cortex, amygdala, superior temporal sulcus, occipital lobe and fusiform gyrus are the key regions of the social brain circuits (Amaral et al., 2008), and categorized into areas related to social impairment, communication deficits, and repetitive behaviors. Compared with healthy controls, ASD patients performed a lower accuracy of semantic sentence comprehension task, and showed a reduced activation in Broca’s area, which involves in expressive language function, while an increased activation in Wernicke’s area, which is essential for receptive language function (Just et al., 2004). Harris et al. examined lexical semantic processing tasks and found a reduced activation in the frontal lobe, whereas a rise activation in the middle temporal gyrus (MTG) in ASD relative to controls (Harris et al., 2006). In addition, compared with TD children, there are significant differences in areas related to speech processing of ASD children aged 2–3 years (Redcay and Courchesne, 2008). Children with autism and low verbal and cognitive performance also showed decreased connectivity within the default, salience, auditory, and frontoparietal networks and decreased inter hemispheric connectivity (Gabrielsen et al., 2018). However, the pattern of brain activity and connectivity changes induced by ASD with language retardation is still unclear. Moreover, the fMRI study on LDD is rare, brain activity differences conducted by the direct comparison of ASD and LDD needs to be explored.

Resting-state functional magnetic resonance imaging (rs-fMRI) is a non-invasive technique to detect spontaneous activity of the human brain at rest, reflecting disease characteristics (Biswal, 2012). Existing studies have focus on static brain activity changes in ASD, there is increasing evidence of the dynamic nature of brain activity and connectivity over time (Allen et al., 2014). Dynamic characteristics of brain activity are related to cognitive function, brain development (Faghiri et al., 2018), and mental disorders (Hutchison et al., 2013; Cui et al., 2020). Dynamic FC method is also applied in children with ASD, and reports that dFC instability between the posterior cingulate gyrus and pars opercularis of the inferior frontal gyrus is associated with dysfunction of social cognitive processes (Li et al., 2020). However, the dynamic characteristics of regional activity in ASD and LDD is still unknown.

Regional homogeneity (ReHo) reflects the local synchronization of a given voxel and its nearest neighbor (Zang et al., 2004). The amplitude of low-frequency fluctuation (ALFF) reflects the power in the effective frequency range (0.01–0.08 Hz) to detect the regional intensity of spontaneous fluctuations and presenting spontaneous brain activity (Zang et al., 2007). Combining ReHo/ALFF and the “sliding window” method, dynamic ReHo (dReHo) and dynamic ALFF (dALFF), calculating the variance of ReHo and ALFF, reflect dynamic changes of spontaneous brain activity over time (Arbabshirani et al., 2017). The dReHo and dALFF method are sensitive approaches for investigating dynamic brain activity (Liao et al., 2019). The decreased temporal variability of dReHo and dALFF indicates lower flexibility of these cerebral neural activities.

We hypothesized that children aged 1–3 years with ASD or LDD would have different brain dynamic abnormalities related to the characteristics of their diseases. Therefore, in this study, we adopted a sliding window method combined with dReHo and dALFF approaches to investigate different dynamic local brain activities between children with ASD and LDD aged 1–3 years, and then further explored its correlation with clinical assessment. We also assessed the accuracy of the dReHo/dALFF alterations in distinguishing ASD from LDD using receiver operating characteristic (ROC) analysis. The aim of this study was to provide a potential biomarker for the early discrimination of toddlers with ASD from those with LDD.

## 2. Materials and methods

### 2.1. Study participants

This study was approved by the Ethics Committee of Hubei Maternal and Child Health Hospital, and informed consent was obtained from all parents prior to the study.

Children aged 1–3 years who visited the Department of Child Health Care of Hubei Maternal and Child Health Hospital because of language impairment were recruited. We examined 58 toddlers with ASD presenting language delay (40 males, 18 females; age 19–36 months) and 36 toddlers with LDD (25 males, 11 females; age 16–36 months). The two groups were well-matched in terms of age, sex, and handedness. Diagnosis of ASD in toddlers was conducted by two experienced neuropsychologists using the Childhood Autism Rating Scale (CARS) and Diagnostic and Statistical Manual of Mental Disorders (DSM-V). The following inclusion criteria were adopted for the ASD group: (1) met the diagnostic criteria of the DSM-V (Zulauf Logoz, 2014; Mukherjee, 2020), (2) CARS score of ≥30. Patients with epilepsy, cerebral palsy, other organic brain diseases, or neurodevelopmental disorders were excluded. None of the individuals with ASD had any known genetic syndromes associated with ASD (e.g., fragile X syndrome).

For the LDD group, the following inclusion criteria were adopted: (1) toddlers with language problems; (2) Gesell developmental schedule with a language development quotient (LDQ) < 75. The exclusion criteria included ASD, hearing impairment, dysarthria, and other related organic diseases.

### 2.2. Behavior measurements

The Autism Behavior Checklist (ABC) as used to evaluate the ASD symptoms. This scale has five subscales: sensory, relaxation, body consciousness, language, and social self-help. All ASD toddlers exhibited an ABC score of at least 68 (average ABC score 83.3 ± 7.0) and presented clinical symptoms of stereotypes, communication disorders, and/or language regression.

The LDQ was measured using the Gesell Scale for Children, which included five subtests, including domains of adaptability, gross motor, fine motor, language and social-emotional responses. The development quotient (DQ) of each domain were calculated for each participant. According to the full-scale DQ, the development of infants was classified as follow: normal (DQ ≥ 85), deficient (DQ < 75), and borderline (75 ≤ ∼ < 85). DQ in any single domain falling below 75 was also considered as deficient within this field.

### 2.3. Magnetic resonance imaging protocol

Thirty minutes before the scanning, all children were sedated by oral administration of 10% chloral hydrate (0.5 ml) and then underwent MRI scanning while falling asleep. A 3.0 T MRI scanner (United Imaging u780) with 8-channel head coil was used. The BOLD-fMRI EPI scan parameters included TR/TE, 2000/30 ms; flip angle, 90°; matrix, 64 × 64; FOV, 240 × 240 mm^2^; thickness/gap, 3/0 mm; slices, 40; scan time, 8 min. In total, 240 scans were collected. The high-resolution anatomic 3D T1 sequence had the following parameters: 176 sagittal slices; TR/TE/TI, 1900/2.1/900 ms; flip angle, 9°; matrix, 256 × 256; FOV, 240 × 240 mm^2^; and thickness/gap, 1/0 mm. This session lasted for 4 min and 26 s. A total of 180 scans were obtained. All participants were scanned for T2 and T2-FLAIR images to exclude obvious brain lesions.

### 2.4. Data analysis

The Data Processing and Analysis of Brain Imaging (DPABI) Toolbox^2^ running on MATLAB 2018b (MathWorks, Natick, MA, USA) was used to preprocess fMRI and structural images in the resting state. It included the following steps: (1) the first 10 time points were removed, and the remaining 230 consecutive volumes were used for data analyses; (2) slice timing correction and head motion correction were performed. The participants with head movements greater than 3 mm and rotation greater than 3 were excluded. In addition, in order to reduce the possible noise caused by head motion, the volume framewise displacement (FD) was taken as the head motion parameter, and participants whose FD exceeded 0.5 were excluded (Jenkinson et al., 2002). Therefore, 3 cases of ASD and 5 cases of LDD were excluded. Spatial normalization to the Montreal Neurological Institute (MNI) space by using infant brain template for zero, four, and five years, and all images were then resampled into 3 mm^3^ resolutions.

For the dynamic ReHo calculations, band-pass filtering (0.01–0.08 Hz) was applied to reduce the low-frequency drift and high-frequency noise. For specificity, a band-pass filter was applied only to the ReHo.

For the dynamic ALFF calculations, the following steps were sequentially performed: spatial smoothing using a 6 mm full-width-at-half-maximum (FWHM) Gaussian kernel, detrending and regressing nuisance covariates such as the head motion effect using Friston-24 model (Friston et al., 1996), white matter signals, cerebrospinal fluid signals, and global signal. Although global signal regression (GSR) remains highly debated, in this study, GSR was utilized because fMRI global signal includes non-neuronal confounds, such as and physiological artifacts, the application of GSR can exclude the influences from these confounds (Murphy and Fox, 2017).

### 2.5. Dynamic measurements

Dynamic local metric analysis was performed using the Temporal Dynamic Analysis (TDA) Toolbox. The sliding window method, which is sensitive to time-dependent changes and whole brain metrics variability, was used to calculate dynamic metrics. The window length is the most critical parameter in resting-state dynamic computation (Liao et al., 2019). Previous studies demonstrated that the minimum window length should be greater than 1/fmin (where fmin is the minimum frequency of the time series) to eliminate spurious fluctuations (Leonardi and Van De Ville, 2015). Therefore, we applied a sliding window length of 50 TR (100 s) and a shift step of 1 TR (2 s). This process generated 181 windows for each participant. Based on these sliding windows, we put forward two local metrics: dALFF and dReHo.

dALFF calculation: After calculating the ALFF of all voxels in the time windows, 18l window-based ALFF maps were obtained for each participant. Then, we calculated the mean and standard deviation (SD) of each voxel in all window-based ALFF maps for each participant, and obtained the corresponding coefficient of variation (CV = SD/mean). Finally, CV maps were smoothed by a 6 mm FWHM Gaussian kernel and prepared for further statistical analysis.

dReHo calculation: Individual ReHo maps were generated by calculating the Kendall coefficient of concordance (KCC) of the time courses of a given voxel with those of its neighbors (26 voxels) in a voxel-wise manner. Then, for each participant, we calculated the CV of each voxel in all window-based ReHo maps. Finally, spatially smoothed CV maps with an isotropic Gaussian kernel of 6 mm FWHM were used for further statistical analyses.

### 2.6. Statistical analysis

The Statistical software SPSS 22.0 (SPSS Inc., Chicago, IL, USA) was used for all analyses. Age and clinical characteristics of toddlers with ASD and LDD were compared using a two-sample *t*-test. Sex differences were determined using Pearson’s chi-square test. With age, gender, and the total intracranial volume (TIV) as covariates, a two-sample *t*-test was applied to compare the dynamic metrics (dALFF and dReHo) of regional brain activity between toddlers with ASD and those with LDD. False discovery rate (FDR) correction was used for multiple comparisons with two-tailed, voxel level *P* < 0.05 as statistically significant.

### 2.7. Correlation analysis with behavior variables

The relationship between dynamic metrics and ABC and CARS scores were conducted by Pearson correlation analysis, with *P* < 0.05 considered statistically significant.

### 2.8. Receiver operating characteristic curve

We hypothesized that differences in dReHo and dALFF between the ASD and LDD groups could be useful diagnostic markers. ROC curves were used to analyze the average values of dReHo and dALFF in various brain areas.

### 2.9. Verification analysis

To validate our dALFF and dReHo findings, three different window lengths [40 TRs (80 s), 50 TRs (100 s), and 60 TRs (120 s)] were calculated in the validation analysis.

## 3. Results

### 3.1. Demographic and behavior measurements

As shown in Table 1, there were no significant differences in sex or age between the ASD and LDD groups. No significant lesions were found in the ASD and LDD groups on conventional MRI. The scores on the ABC and CARS scales in the ASD group were significantly higher than those in the LDD group (*P* < 0.001).

**TABLE 1:** Participant characteristics discriminated according to autism spectrum disorder (ASD) or language developmental delays (LDD).

Characteristics	ASD (*n* = 55)	LDD (*n* = 31)	*P*-value
Sex (Male/Female)	37/18	21/10	0.964
Age (months, Mean ± SD)	30 ± 4	29 ± 6	0.438
LDQ score	63 ± 7.0	66 ± 6.0	0.053
ABC score	83.3 ± 7.0	35 ± 5.8	<0.001*
CARS score	34.8 ± 3.2	15 ± 2.1	<0.001*

ASD, autism spectrum disorder; LDD, language development delay; LDQ, language development quotient; ABC, Autism Behavior Checklist; CARS, Childhood Autism Rating Scale.

*Represents statistical significant.

Bold values represents statistical significant.

### 3.2. dReHo differences

**FIGURE 1:**
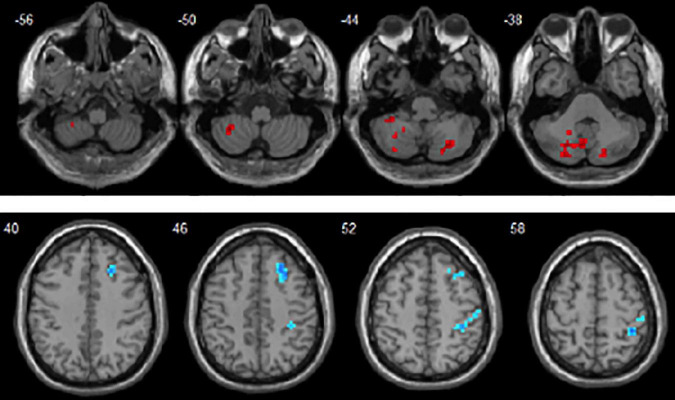
Brain areas with significant dReHo differences between autism spectrum disorder (ASD) and language development delay (LDD) groups. Regions in red-yellow are brain areas where dReHo was significantly increased in ASD compared to LDD. Regions in blue-green are brain areas where dReHo was significantly decreased in ASD compared to LDD. The results were multiple compared at the voxel-level (*P* < 0.05, FDR corrected). The left side of the image corresponds to the left hemisphere of the brain.

Compared with the LDD group, the ASD group showed increased dReHo in the left cerebellum_8/Crust2 and the right cerebellum_Crust2, and decreased dReHo in the right middle frontal gyrus (MFG) and the right post-central gyrus (Table 2 and Figure 1).

**TABLE 2:** Brain regions showing significant dynamic regional homogeneity (dReHo) differences between autism spectrum disorder (ASD) and language developmental delays (LDD) groups.

Specific effects	Identified brain regions	Peak coordinates (MNI)			Side	Peak T	Cluster size (voxels)
		X	Y	Z			
**ASD > LDD**							
	Cerebellum_8/Crust2	−33	−51	−51	L	3.45	139
	Cerebellum_Crust2	12	−78	−30	R	3.74	75
**ASD < LDD**							
	Middle frontal gyrus	24	27	45	R	−4.10	78
	Post-central gyrus	39	−36	54	R	−4.20	77

Voxel-level *P* < 0.05 corrected for false discovery rate. ASD, autism spectrum disorder; LDD, language development delay; MNI, Montreal Neurological Institute; dReHo, dynamic regional homogeneity.

### 3.3. dALFF differences

**FIGURE 2:**
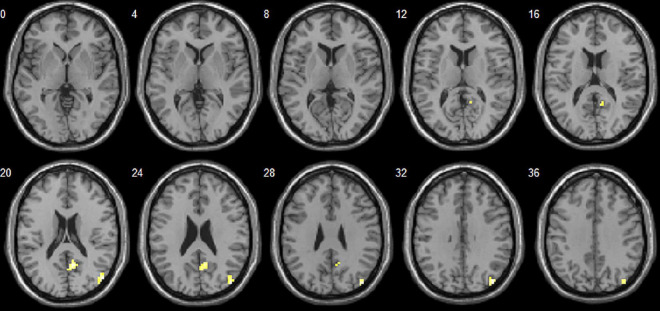
Brain areas with significant dALFF differences between autism spectrum disorder (ASD) and language development delay (LDD) groups. Regions in red-yellow are brain areas where dALFF was significantly decreased in ASD compared to LDD. The results were multiple compared at the voxel-level (*P* < 0.05, FDR corrected). The left side of the image corresponds to the left hemisphere of the brain.

Toddlers with ASD also exhibited decreased dALFF values in the right middle temporal gyrus (MTG) and right precuneus (Table 3 and Figure 2).

**TABLE 3:** Brain regions showing significant dynamic amplitude of low-frequency fluctuation (dALFF) differences between autism spectrum disorder (ASD) and language developmental delays (LDD) groups.

Specific effects	Identified brain regions	Peak coordinates (MNI)			Side	Peak T	Cluster size (voxels)
		X	Y	Z			
**ASD < LDD**							
	Middle temporal gyrus	42	−81	30	R	−4.27	52
	Precuneus	9	−57	21	R	−4.10	39

Voxel-level *P* < 0.05 corrected for false discovery rate. ASD, autism spectrum disorder; LDD, language development delay; MNI, Montreal Neurological Institute; dALFF, dynamic amplitude of low-frequency fluctuation.

### 3.4. Correlation with behavior variables

**FIGURE 3:**
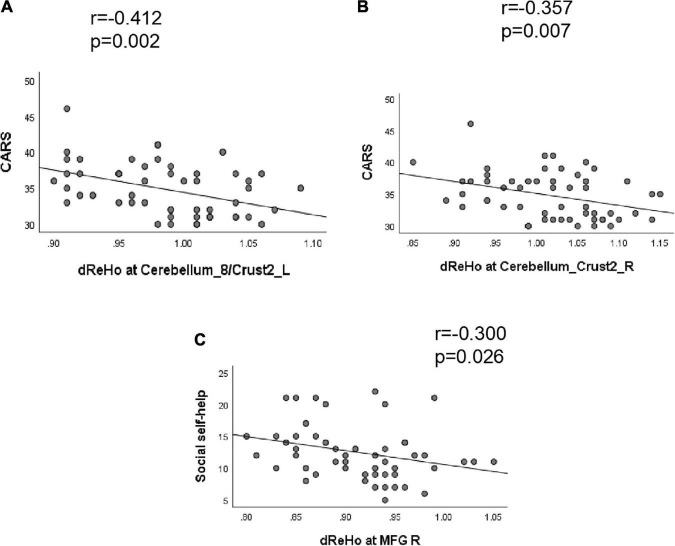
Correlation between dReHo value and behavioral scores (ABC/CARS) in autism spectrum disorder (ASD) group. **(A)** The CARS score was negatively correlated with the dReHo of the left cerebellum_8/crust2 (*r* = –0.412, *P* = 0.002). **(B)** The CARS score was negatively correlated with the dReHo of the right cerebellum_crust2 (*r* = –0.357, *P* = 0.007). **(C)** The dReHo value of the right MFG were negatively correlated with the social self-help of the ABC score (*r* = –0.300, *P* = 0.026).

Correlation analysis showed that the CARS score was negatively correlated with the dReHo of the left cerebellum_8/crust2 (*r* = −0.412, *P* = 0.002) and right cerebellum_crust2 (*r* = −0.357, *P* = 0.007). The dReHo value of the right MFG was negatively correlated with social self-help of the ABC score (*r* = −0.300, *P* = 0.026). No significant correlation was observed between the dALFF value and the ABC or CARS scores (Table 4 and Figure 3).

**TABLE 4:** Correlation between dynamic regional homogeneity/dynamic amplitude of low-frequency fluctuation (dReHo/dALFF) values and behavioral scores Autism Behavior Checklist/Childhood Autism Rating Scale (ABC/CARS) in the autism spectrum disorder (ASD) group.

	Identified brain regions	ABC						CARS
		Total	Sensory	Relating	Body concept	Language	Social self-help	
dReHo	Cerebellum_8/crust2_L							
	*r*	−0.082	−0.013	0.140	−0.102	−0.108	−0.149	−0.412
	*p*	0.553	0.927	0.308	0.460	0.433	0.278	0.002*
	Cerebellum_crust2_R							
	*r*	0.016	0.016	0.266	−0.102	−0.017	−0.218	−0.357
	*p*	0.908	0.906	0.050	0.46	0.905	0.109	0.007*
	Middle frontal gyrus_R							
	*r*	0.099	0.124	0.134	0.128	0.351	−0.300	0.026
	*p*	0.471	0.366	0.331	0.351	0.464	0.026*	0.178
	Post-central gyrus_R							
	*r*	−0.194	−0.096	−0.211	−0.025	0.056	−0.117	0.167
	*p*	0.156	0.484	0.122	0.854	0.683	0.396	0.222
dALLF	Middle temporal gyrus_R							
	*r*	−0.031	0.102	−0.086	−0.071	0.175	−0.176	−0.076
	*p*	0.823	0.460	0.531	0.606	0.200	0.918	0.580
	Precuneus_R							
	*r*	0.037	0.006	0.039	−0.220	0.224	−0.008	0.100
	*p*	0.789	0.963	0.775	0.107	0.102	0.954	0.468

*P < 0.05.

ASD, autism spectrum disorder; LDD, language development delay; MNI, Montreal Neurological Institute; dReHo, dynamic regional homogeneity; dALFF, dynamic amplitude of low-frequency fluctuation; ABC, Autism Behavior Checklist; CARS, Childhood Autism Rating Scale; L, left; R, right.

Bold values represents statistical significant.

### 3.5. ROC curve

**FIGURE 4:**
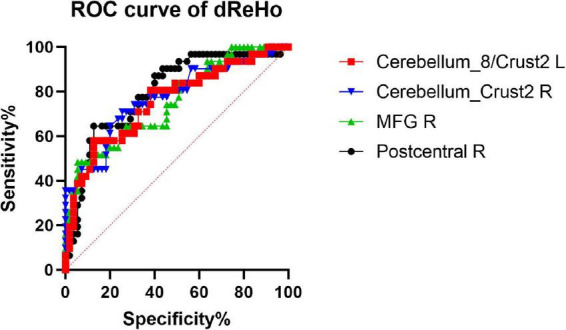
Receiver operating characteristic (ROC) curve analysis of the mean dReHo values for altered brain regions. The AUCs for dReHo values were as follows: cerebellum_8/crust2_L, 0.76; cerebellum_crust2_R, 0.77; MFG_R, 0.75; post-central gyrus, R, 0.80. MFG_R: right middle frontal gyrus; AUC: area under the curve.

**FIGURE 5:**
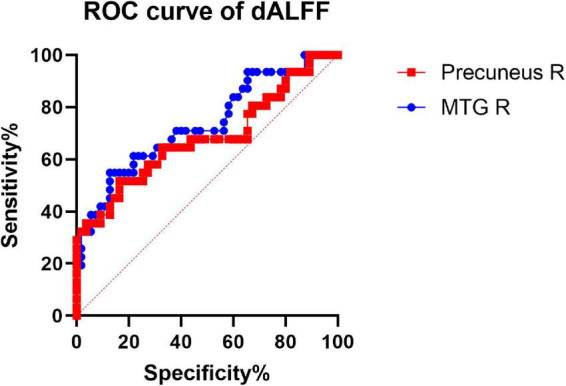
Receiver operating characteristic (ROC) curve analysis of the mean dALFF values for altered brain regions. The AUCs for dALFF values were Precuneus_R: 0.68. MTG_R: right middle temporal gyrus; AUC: area under the curve.

We hypothesized that the dReHo and dALFF differences between the two groups might be useful diagnostic markers. The mean dReHo and dALFF values in different brain regions were analyzed using the receiver operating characteristic (ROC) curves. When the area under the curve (AUC) is 0.5–0.7 it indicates accuracy is low, if it is 0.7–0.9 accuracy is certain. The AUCs for dReHo values were as follows: cerebellum_8/crust2_L (0.76, *P* < 0.0001, sensitivity 58.06%, specificity 87.27%), cerebellum_crust2_R (0.77, *P* < 0.0001, sensitivity 70.97%, specificity 74.55%), right MFG (0.75, *P* = 0.0001, sensitivity 48.39%, specificity 94.55%), and right post-central gyrus (0.80, *P* < 0.0001, sensitivity 64.52%, specificity 87.27%) (Figure 4). Moreover, the AUCs for the dALFF values were as follows: right MTG (0.73, *P* = 0.003, sensitivity 54.84%, specificity 87.27%), and right precuneus (0.68, *P* = 0.005, sensitivity 51.61%, specificity 83.64%) (Figure 5).

### 3.6. Verification analysis

By using three different window lengths [40 TRs (80 s), 50 TRs (100 s), and 60 TRs (120 s)] with two window steps [1 TR (2 s) and 4 TR (8 s)], we found that the differences in dReHo and dALFF between ASD and LDD were similar to our main findings. These results were provided in Supplementary Figures 1–8.

## 4. Discussion

In this study, dReHo and dALFF were used to investigate the differences in temporal variability of regional brain activity between toddlers with ASD and those with LDD. Compared with the LDD group, the ASD group showed increased dReHo in the left cerebellum_8/Crust2 and right cerebellum_Crust2, and decreased dReHo in the right MFG and right post-central gyrus. Patients with ASD also exhibited decreased dALFF values in the right MTG and right precuneus. Moreover, correlation analysis showed that the CARS score was negatively correlated with the dReHo of the left cerebellum_8/crust2 and right cerebellum_crust2. Moreover, the dReHo value of the right MFG was negatively correlated with social self-help based on the ABC score. These findings suggest that dynamic regional activity might be applied in clinical practice in the future to distinguish ASD from LDD.

Many neuroimaging studies have proposed hypotheses regarding the cortical basis of ASD with language dysfunction, one of which is the reversed functional lateralization of the language network (Herringshaw et al., 2016). Both the Broca (located in the left inferior frontal gyrus) and Wernicke (located in the left superior temporal gyrus) areas, which mediate social language processing, are located in the left hemisphere (Kana and Wadsworth, 2012). In ASD, the Broca and Wernicke areas are regarded as functionally impaired. However, compared with TD children, children with ASD show significantly stronger right hemisphere activation in speech perception tasks (Redcay and Courchesne, 2008). For example, rs-fMRI studies have found decreased ReHo in the right superior temporal sulcus, right inferior frontal gyrus (IFG), right middle frontal gyrus (MFG), right post-central gyrus, right insula, and bilateral cerebellum crust1 in ASD (Paakki et al., 2010). Our research also found decreased dReHo in the MFG and post-central gyrus and decreased dALFF in the MTG and precuneus on the right side, relative to LDD. Therefore, it is reasonable to infer those children diagnosed with ASD at the age of 1–3 years not only have impaired left language brain function but also have more extensive functional connectivity abnormalities in the right language brain region compared to children with LDD alone, which may provide certain clues for the early differential diagnosis of ASD and LDD.

The prefrontal cortex is the core area for ASD. Functional imaging studies have shown that the prefrontal cortex plays an important role in social cognition (Forbes and Grafman, 2010). Research on early brain growth in children with ASD showed increased gray matter volume in the prefrontal cortex, including the orbitofrontal cortex, IFG, MFG, and SFG. This is one of the reliable findings in ASD, and its pathological basis was found to be a significantly increased number of neurons (Courchesne et al., 2011). However, rs-fMRI studies have described an extensively weakened prefrontal-posterior functional connectivity, which may indicate impairment of cognitive, social, and speech processing in ASD. Enticott et al. found that repetitive transcranial magnetic stimulation (rTMS) to the medial prefrontal cortex can reduce social-related impairment in patients with ASD, indicating that this may be a target region for these symptoms (Enticott et al., 2014). Consistently with previous rs-fMRI studies, we found a reduced dReHo in the right MFG. Further correlation analysis showed that the dReHo value of the right MFG in ASD children correlated with social self-help assessed via the ABC score. This indicates that the worse the local consistency of the right MFG brain region, the higher the social and self-help scores on the ABC scale, and the worse the social and self-help ability. Studies have found that the right MFG is related to high-level executive function. For example, attention, memory, planning, and judgment (Talati and Hirsch, 2005) and the decreased dReHo value of the right MFG will affect the social self-care ability of children with ASD, which is consistent with the results of this study. It also suggests that compared with children with LDD alone, the early social and self-help ability of children with ASD needs special attention. The ROC curve showed 48.39% sensitivity and 94.55% specificity to distinguish ASD from LDD. Therefore, these findings confirm the importance of the prefrontal cortex in the early diagnosis of ASD.

In addition to language-related activities and processes (McDermott et al., 2003; Whitney et al., 2011; Hickok, 2012), the MTG is also involved in action observation (Whitney et al., 2011), situational investigation (Hsu and Sonuga-Barke, 2016), complex sound processing (Hesling et al., 2005), logical reasoning (Goel et al., 1998), and dynamic facial expression recognition (Sato et al., 2012). Recently, more and more researchers have expressed the opinion that abnormalities in the “social network” (Adolphs, 2003; Blakemore, 2012) may be the neural mechanism underlying social dysfunction in ASD (Pelphrey and Carter, 2008). As one of the main nodes of the “social network” (Sato et al., 2017), MTG abnormalities have been reported many times in ASD (Assaf et al., 2013; Yerys et al., 2015; Ogawa et al., 2019). Our study also found reduced ALFF variability in the right MTG in ASD patients, which is consistent with local connectivity reduction (Itahashi et al., 2015) and morphological changes (Libero et al., 2014). Compared with LDD, the right MTG may be more related to the pathogenesis of social dysfunction, which is the core symptom of ASD.

The precuneus is a hub in the default mode network (DMN). The clinical manifestations of ASD overlap with the functions of the DMN, including emotional processing, perception of interpersonal communication, and judgment of self and others’ mental states; suggesting that the DMN may be damaged in ASD (Glerean et al., 2016). Although functional connectivity (FC) in the DMN is aberrant, either increased or decreased FC has been reported in ASD patients. We found decreased dALFF in the precuneus in ASD compared to LDD, providing additional evidence of DMN defects.

The cerebellum is one of the most common regions presenting abnormalities in ASD, and this has been variously reported from the cellular to the behavioral level. Most postmortem studies on ASD have reported reduced Purkinje cell counts in the cerebellar cortex, and specific targeting of cerebellar Purkinje cells induces ASD-related symptoms in animal models (Di Martino et al., 2014). Structural differences in the ASD cerebellum are associated with social and communication impairments, limited interest, and repetitive behaviors. At the genetic level, genes involved in ASD (e.g., SHANK3, EN2, and RORA) are commonly associated with cerebellar development (Rogers et al., 2013). The current results found that toddlers with ASD exhibited significantly higher dReHo values in the left cerebellum_8/Crust2 and the right cerebellum_Crust2 than those with LDD, with a negative correlation with CARS score. These results indicate that cerebellar development is abnormal in early ASD compared with LDD alone, and this abnormality is directly related to the severity of ASD. Considered together, these pieces of evidence led us to speculate that the cerebellum may regulate the dysfunction of the neocortical circuit by enhancing its own function, which might be a potential mechanism that explains some aspects of the ASD phenotype (Hegarty et al., 2018).

This study had some limitations. First, the heterogeneity of autism suggests that further studies and analysis of subtypes may lead to clearer imaging markers. Secondly, a recent area of development in ASD research is the classification of ASD based on resting-state data in conjunction with machine learning and deep learning techniques. In the future, by using dynamic brain parameters as inputs, we expect that the machine learning classifier will show a high level of accuracy.

In summary, the clinical manifestations of ASD with language delay in the early stages among toddlers are like those of LDD and are difficult to distinguish by clinical evaluation alone. The current study suggests the presence of decreased dReHo/dALFF in the right MFG, post-central gyrus, and MTG, and increased dReHo in the bilateral cerebellum in ASD relative to LDD. Our results may be helpful for the diagnosis of ASD. Identifying the differences in brain development between ASD and LDD will be the first step in the development of novel early screening tools, the determination of when to treat, and the evaluation of the intervention efficacy in ASD. Our next step is to follow up with the children with autism so that we can conduct a pre- and post-control study.

## Data availability statement

The raw data supporting the conclusions of this article will be made available by the authors, without undue reservation.

## Ethics statement

The studies involving human participants were reviewed and approved by the Ethics Committee of Hubei Maternal and Child Health Hospital. Written informed consent to participate in this study was provided by the participants’ legal guardian/next of kin.

## Author contributions

SW: experimental design, data collection, data analysis, and article writing. ZW: data processing and analysis and article writing. HW: experimental design, data collection, article writing, and funds to support. FL: data processing and article writing. WY and CJ: data collection. YZ and ZZ: article writing. AZ and YW: data collection and statistical analysis. XL and XW: data processing and analysis and literature review. All authors contributed to the article and approved the submitted version.

## Footnotes

1
https://www.autismspeaks.org/autism-statistics-asd

2
http://www.rfmri.org/dpabi
