# Supplementary material for: Potential dynamic regional brain biomarkers for early discrimination of autism and language development delay in toddlers
Source: Front Neurosci. 2023 Jan 9;16:1097244. doi: 10.3389/fnins.2022.1097244 (PMC9869111; doi:10.3389/fnins.2022.1097244)
Supplement: Supplementary file 1 [file Data_Sheet_1.docx]

**
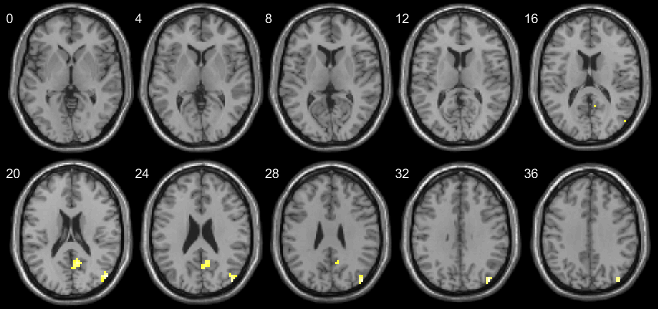
Figure S1 Brain areas with significant dALFF differences derived from 40 TRs (80s)**

**window size and 1 TR (2s) step between ASD and LDD groups**


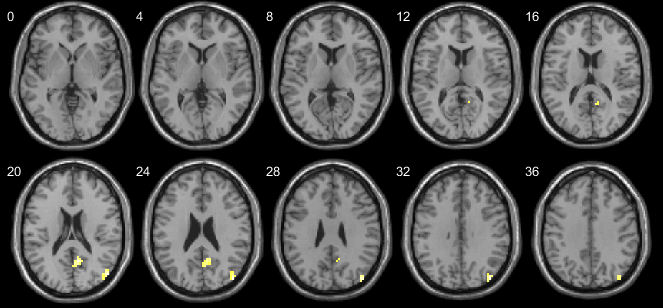


**Figure S2 Brain areas with significant dALFF differences derived from 50 TRs (100s)**

**window size and 1 TR (2s) step between ASD and LDD groups**


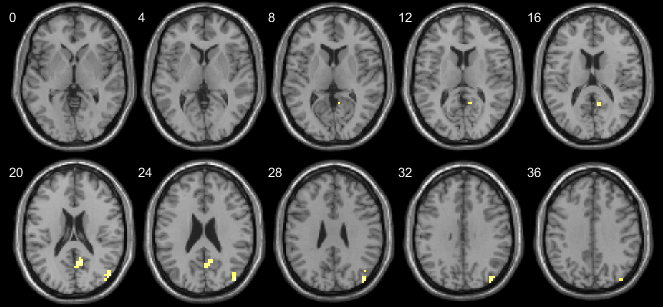


**Figure S3 Brain areas with significant dALFF differences derived from 60 TRs (120s)**

**window size and 1 TR (2s) step between ASD and LDD groups**


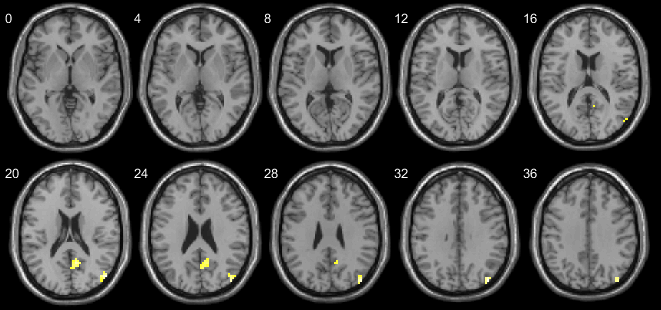


**Figure S4 Brain areas with significant dALFF differences derived from 40 TRs (80s)**

**window size and 4 TRs (8s) step between ASD and LDD groups**


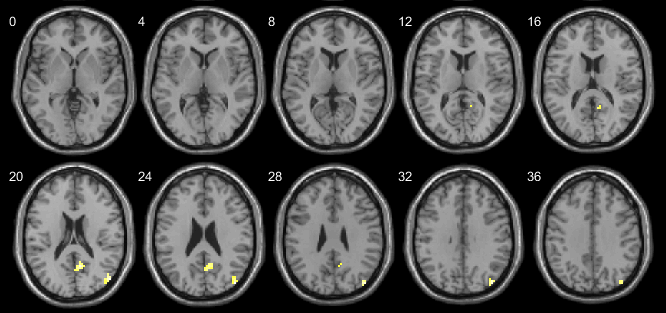


**Figure S5 Brain areas with significant dALFF differences derived from 50 TRs (100s)**

**window size and 4 TRs (8s) step between ASD and LDD groups**


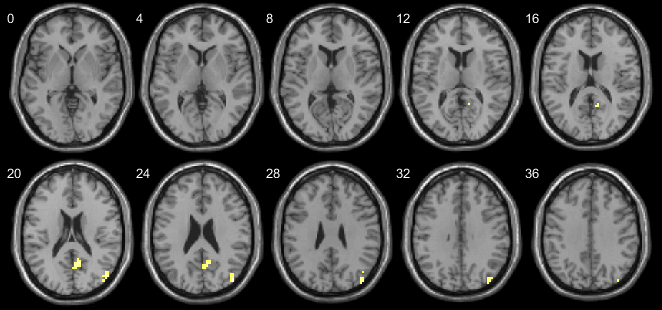


**Figure S6 Brain areas with significant dALFF differences derived from 60 TRs (120s)**

**window size and 4 TRs (8s) step between ASD and LDD groups**


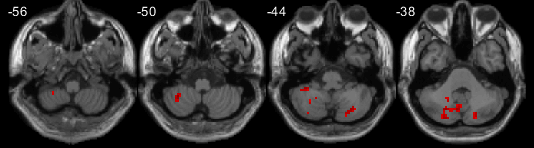

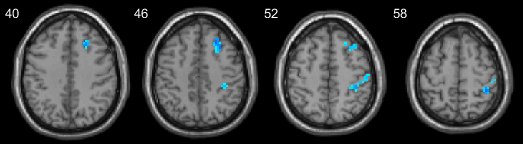


**Figure S7 Brain areas with significant dReHo differences derived from**

**40 TRs (80s) window size and 1 TR (2s) step between ASD and LDD groups**


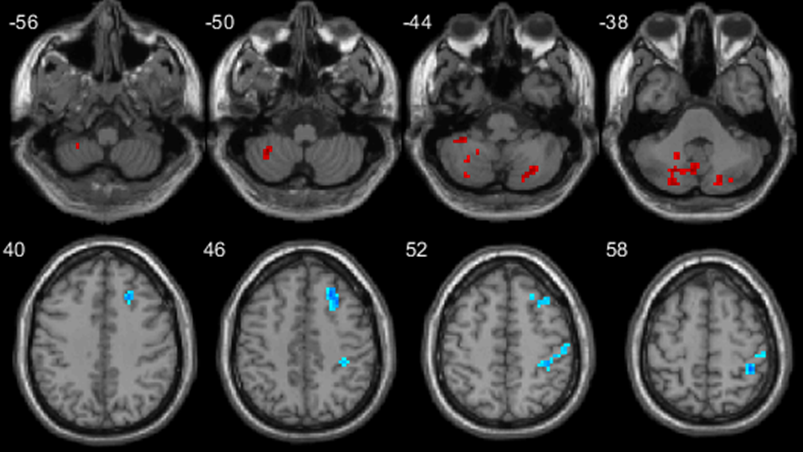


**Figure S8 Brain areas with significant dReHo differences derived from**

**50 TRs (100s) window size and 1 TR (2s) step between ASD and LDD groups**


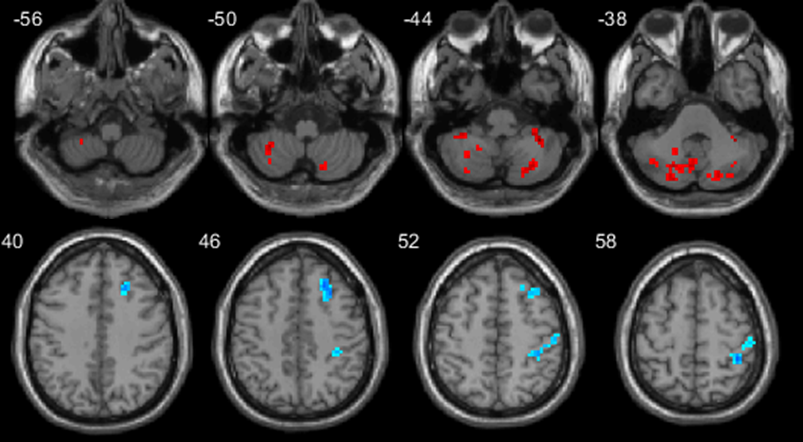


**Figure S9 Brain areas with significant dReHo differences derived from**

**60 TRs (120s) window size and 1 TR (2s) step between ASD and LDD groups**
